# Benefit finding in adults living with somatic non-communicable chronic disease: a systematic review on the mean level and the prevalence

**DOI:** 10.3389/fpsyt.2025.1452218

**Published:** 2026-01-26

**Authors:** Zhunzhun Liu, Xiang Hu, Volker Arndt, Melissa S. Y. Thong

**Affiliations:** 1 Nursing School, Wuxi Taihu University, Wuxi, Jiangsu, China; 2 Unit of Cancer Survivorship, German Cancer Research Center (DKFZ), Heidelberg, Germany; 3 The Key Research Institute of Chongqing for Curriculum & Instruction, School of Education, Chongqing Normal University, Chongqing, Germany

**Keywords:** benefit finding, adult, chronic illness, non-communicable chronic disease, systematic review

## Abstract

**Introduction:**

Benefit finding (BF) refers to the phenomenon of identifying positive changes from challenging circumstances. This review aimed to comprehensively assess the BF prevalence in persons with somatic non-communicable chronic diseases (pSNCDs).

**Materials and methods:**

Electronic databases were systematically searched using the concept “benefit finding”. Eligible studies (adult persons with SNCDs, BF measured by the Benefit Finding Scale, written in English, observational in design, and published until June 30, 2023) were reviewed and underwent quality and publication bias assessments; prevalence and mean were extracted and synthesized.

**Results:**

Of the 55 included articles, 35 were rated “weak”, 18 “moderate”, and 2 “strong” in quality. BF mean was 3.25 (25,972 observations), BF prevalence was 97.5% (10,720 observations), and moderate-to-high BF prevalence was 74.1% (12,363 observations). Subgroup analysis indicated higher moderate-to-high BF in cancer survivors (highest: 89% in breast cancer) than non-cancer survivors (highest: 72.4% in HIV). The lowest BF and moderate-to-high BF prevalence occurred in cancer survivors aged 60 +. Meta-regression suggested that study design and quality, time since diagnosis, gender, and mean age may explain heterogeneity in BF means. Subgroup analyses showed the lowest BF mean in pSNCDs aged 70+ years.

**Discussion:**

Persons with SNCDs generally reported a moderate BF mean. Age is an important factor associated with BF. Future studies are needed to better understand how pSNCDs find/perceive benefits, especially for older adults aged 60+ years.

**Systematic review registration:**

https://www.crd.york.ac.uk/PROSPERO/view/CRD42022308513, identifier CRD42022308513.

## Introduction

Somatic non-communicable chronic diseases (SNCDs) are long-lasting diseases (excluding mental illness) that may develop through unhealthy lifestyles (e.g., tobacco use, excessive alcohol consumption, lack of physical activity, and poor diet), degenerative changes, or family genetics (1). Cardiovascular diseases, cancers, chronic pulmonary diseases, and diabetes are the main disease clusters of SNCDs that account for over 80% of all premature deaths from SNCDs (2). Deaths resulting from SNCDs account for approximately 74% of all deaths globally in 2019 (2). The global epidemic of SNCDs poses a burden on the healthcare system of all countries, e.g., the short-term and long-term treatment of SNCDs (1). SNCDs and related treatments can be challenging and may negatively affect persons living with SNCDs (pSNCDs), e.g., increasing the risk of disabilities (3) and mental health disorders (4). These detriments can persist indeterminately. However, pSNCDs can also identify positive changes experienced from the SNCD trajectory, which is labeled as benefit finding (BF) or posttraumatic growth.

BF is defined as a form of cognitive adaptation response to adversity via positive evaluations of the circumstances that an individual encounters (5), or the “selective evaluation process [that] minimizes victimization by focusing on … beneficial qualities of the situation” (p. 26) (6). On the one hand, the definition implies that perceived threat posed by an adversity can be reduced through the BF process, but does not imply the intentional denial of its potential harmful aspects, which has been supported by previous observational studies (6, 7). On the other hand, the BF definition also emphasizes the process of constructing benefits from adversity or finding the good in bad events (8). An overview reported that the majority of persons living with chronic diseases experienced benefits or gains from their medical adversity (9). The answers on multiple aspects of changes, e.g., restoring comfort views of themselves, reorganizing their world orderly, and viewing their life as meaningful (9, 10), suggested that BF could be assessed through positive changes that individuals experienced.

Various instruments have been used to assess BF in adult pSNCDs, e.g., the Perceived Benefit Scale, Positive Contribution Scale, and Benefit Finding Scale (BFS). The BFS is the most widely used instrument to measure BF in adult pSNCDs. Antoni et al. developed the original 17-item BFS in patients diagnosed with early-stage breast cancer (7). The BFS is based on the Positive Contribution Scale, which assessed the perceived benefits among parents of children with special needs. The BFS has demonstrated good reliability and validity and has been translated and cross-culturally validated in many countries (11). Although the BFS has been mainly used in cancer populations (6), it has also been implemented in studies covering a wide array of other medical conditions, e.g., rheumatoid arthritis (12) and multiple sclerosis (9).

Although the mean score of the BFS is often calculated for research purposes, it may not be useful to determine the extent of BF (e.g., prevalence) for clinical purposes. To better understand the phenomenon in the target sample, researchers additionally have to determine the cut-offs to describe the percentage of the sample who report BF. A commonly used cut-off is a score of ≥3, dichotomizing the BFS score into none-to-low and moderate-to-high (13, 14). In the study conducted by Jansen et al., 99% colorectal cancer survivors reported at least one benefit (BF prevalence), and 64% had a mean score/item ≥3 (moderate-to-high BF prevalence) (13). Furthermore, BF prevalence can be used to reveal the impact of BF on the target population. By investigating moderate-to-high BF prevalence in 6,952 cancer survivors 5–16 years after diagnosis, a previous study found that 66% reported moderate-to-high BF, and survivors who were older, male, with more advanced cancer, and with fewer years since diagnosis were less likely to report moderate-to-high BF (14). However, when determining BF prevalence, it is critical to consider the selected cut-off to avoid ceiling effects or floor effects.

Unfortunately, there is no consensus on the cut-offs and reporting of BF prevalence (crude prevalence or adjusted prevalence). For instance, one study (15) reported high BF (4 = quite a bit or 5 = extremely) prevalence in survivors with brain tumors; another study (14) reported both moderate-to-high BF (3 = moderately or 4 = quite a bit or 5 = extremely) prevalence, while other researchers (12, 13) also reported BF (experience one or more items) prevalence in their studies. The inconsistencies in the cut-off used to report prevalence make it difficult to synthesize and compare BF prevalence.

## Aims

The aim of this systematic review was to provide a comprehensive overview of the prevalence and mean level of BF measured by the BFS in pSNCDs in current publications.

## Materials and methods

This systematic review is registered in the International Prospective Register of Systematic Reviews (PROSPERO; Register ID: CRD42022308513) and is conducted and reported in line with Preferred Reporting Items for Systematic Reviews and Meta-Analyses (PRISMA) guidelines (16).

### Search strategy

The electronic databases used for the literature search included PubMed (Medline), Web of Science (WOK, CCC, and SciELO), and EBSCOhost research databases (PsycINFO, PsycARTICLES, CINAHL, and PSYNDEX literature with PSYNDEX Tests). Publications (from inception) up to December 31, 2021, were reviewed. Search terms related to “benefit finding”, “adult”, “chronic disease”, and “Benefit Finding Scale” were applied and were iterative by refinements (language, English; sample group, human) to identify studies (**Supplementary Table 1**). Potentially relevant publications were also identified through the reference lists of previously published systematic reviews and included studies. An updated search was performed on June 30, 2023, to retrieve articles published since the first search.

### Eligibility criteria

Studies were included if they examined the prevalence of BF in adult persons with SNCDs (no less than 18 years old), they reported in English, they were observational in design (cross-sectional, case–control, and cohort studies), or BF was operationally defined and measured using the validated BFS. Studies were excluded if 1) they were not published in a peer-reviewed journal, 2) they focused on neurological (e.g., dementia) and substance use disorders, or 3) they were conducted in persons with a history of serious psychiatric illness.

### Study selection

All records retrieved from the search were imported into EndNote X9 (Clarivate Analytics, PA, USA) for duplicate deletion and further checking. One reviewer (Author 1) screened the titles and abstracts to identify articles that met or possibly met the above inclusion criteria. Subsequently, Author 1 further assessed the full text of the selected articles against the eligibility criteria in detail. The reasons for the exclusion of identified articles were recorded. Where necessary, decisions regarding the inclusion and exclusion of studies were discussed and confirmed with another reviewer (Author 2). Author 1 also contacted the first/corresponding author for detailed BF prevalence and BF mean score if these were not available in the included publication. If neither of them was obtained, then an excluding decision was made.

Including repeated results and samples in the meta-analysis can result in underestimating error variance and lead to a misinterpretation of significance tests. The following criteria were used to avoid multiple counting of respondents:

1. If a single study reported more than one illness or two or more articles published results from the same study and data collection period, only results reporting the highest number of cases were used in the analysis of the overall pooled prevalence of BF. However, these cases were pooled for specific illness estimates.
2. If two studies used the same sample but reported results measured in different study time points (one cohort study reported both cohort study results and cross-sectional study results), we coded the one reported as the original study design, the cohort study.
3. If a sample using the same follow-up period was reported in two or more of the included studies, and these studies reported equivalent BF prevalence at baseline, then the earliest published study will be included.
4. If a single study reported BF at more than one time point (longitudinal data), the BF data with the highest number of cases were chosen.

### Methodological quality assessment

Two reviewers (Author 1 and Author 2) independently reviewed the methodological quality of each included study using the tool developed by the *Effective Public Health Practice Project* (EPHPP) (online available from https://www.ephpp.ca/quality-assessment-tool-for-quantitative-studies/). The scoring of the instrument was adapted according to the objectives and inclusion criteria of this review (**Supplementary Table 2**). We assessed the methodological quality of each study in the following six areas: 1) selection bias, 2) study design, 3) confounders, 4) data collection methods, 5) withdrawals and dropouts, and 6) analysis. Each area was rated as weak, moderate, or strong. An overall methodological rating was classified as weak (if two or more weak ratings appear in six areas), moderate (one weak rating), or strong (no weak rating). Potential publication bias was evaluated using funnel plots.

### Data extraction

The two reviewers (Author 1 and Author 2) independently extracted the data from the included articles. If there was a discrepancy that could not be resolved between the two reviewers, a third reviewer (Author 4) was involved. Information extracted from each included study was as follows: 1) basic information of a study, 2) demographic characteristics of the sample, 3) illness characteristics, 4) measurements, 5) prevalence, and 6) mean score.

### Data analysis

To quantitatively present the distribution of the characteristics of the included articles and samples, we reported the means, standard deviations, and ranges, or frequencies and percentages to describe the features of each characteristic.

*Primary outcome: BF prevalence.* According to the Likert-type scale and cut-off value used in the included studies, prevalence was categorized according to the BF prevalence and moderate-to-high BF prevalence categories:

1. *BF prevalence was defined and synthesized according to the following two scenarios:*
*If using a Likert-type scale from 1 (not at all) to 5 (extremely) or a Likert-type scale from 1 (not at all) to 3 (great deal).*
  Definition a: The percentage of participants whose mean item score > 1.
*If using a Likert-type scale from 1 (strongly disagree) to 5 (strongly agree).*
  Definition b: The percentage of participants whose mean item score ≥3.
2. *Moderate-to-high BF prevalence was defined and synthesized according to the following three scenarios:*
*If using a Likert-type scale from 1 (not at all) to 5 (extremely).*
  Definition c: The percentage of participants whose mean item score ≥3 (including the definition given in the included studies: a total average BFS score of >2 indicates at least a moderate amount of perceived BF).
*If using a Likert-type scale from 1 (not at all) to 3 (great deal).*
  Definition d: The percentage of participants whose mean item score >2.
*If using a Likert-type scale from 1 (strongly disagree) to 5 (strongly agree).*
  Definition e: The percentage of participants whose mean item score ≥4.

*Secondary outcome:* mean (M), standard deviations (SDs), and 95% confidence interval (95% CI) ranges.

The mean item scoring (M_item_) was chosen to avoid the impact of the number of items (N_item_) using the following equations: M_item_ = M/N_item_ and SD_item_ = SD/N_item._ For each included study, the versions of the Likert type were transformed into a 5-point Likert-type scoring (from 1 = *not at all* to 5 = *extremely* or from 1 = *strongly disagree* to 5 = *strongly agree*); see **Supplementary Table 3**. When synthesizing subgroup results in one study, transformation was adopted according to **Supplementary Table 4**.

Log transformation and logit transformation were used to transform the prevalence and moderate-to-high prevalence. The pooled prevalence and mean (raw) estimates of BF in adults were calculated using random-effects meta-analysis, accounting for clinical heterogeneity. The I-squared (I^2^) statistic was used to assess heterogeneity across studies; I^2^ > 50% indicated significant heterogeneity. If included studies were ≥10, the influencing factors of heterogeneity were investigated where possible with meta-regression for BF analysis. Factors studied included year of report (2000s, 2010s, and 2020s), study design (longitudinal and cross-sectional), sample frame (single-centered, population-based, and cannot be determined), methodological quality (strong, moderate, and weak), diagnosis (cancer and non-cancer), time since diagnosis (<1 year, 1–5 years, >5 years, and not given), gender (female, male, and both), mean age at survey (≤50, 51–60, 61–70, and >70), geographic origin (location from which participants were sampled), and sample size (<100, 100–300, 301–500 and >500). Subgroup analyses for BF mean and BF prevalence, stratified by cancer and non-cancer, were conducted.

Sensitivity analysis was conducted by omitting each one of the studies from the main analysis to examine whether results would significantly change. The potential publication bias was also assessed using funnel plots combined with tests developed by Egger and Begg. A *p*-value <0.1 in either Egger’s or Begg’s test indicated the presence of publication bias.

## Results

### Literature search results

**Figure 1:**
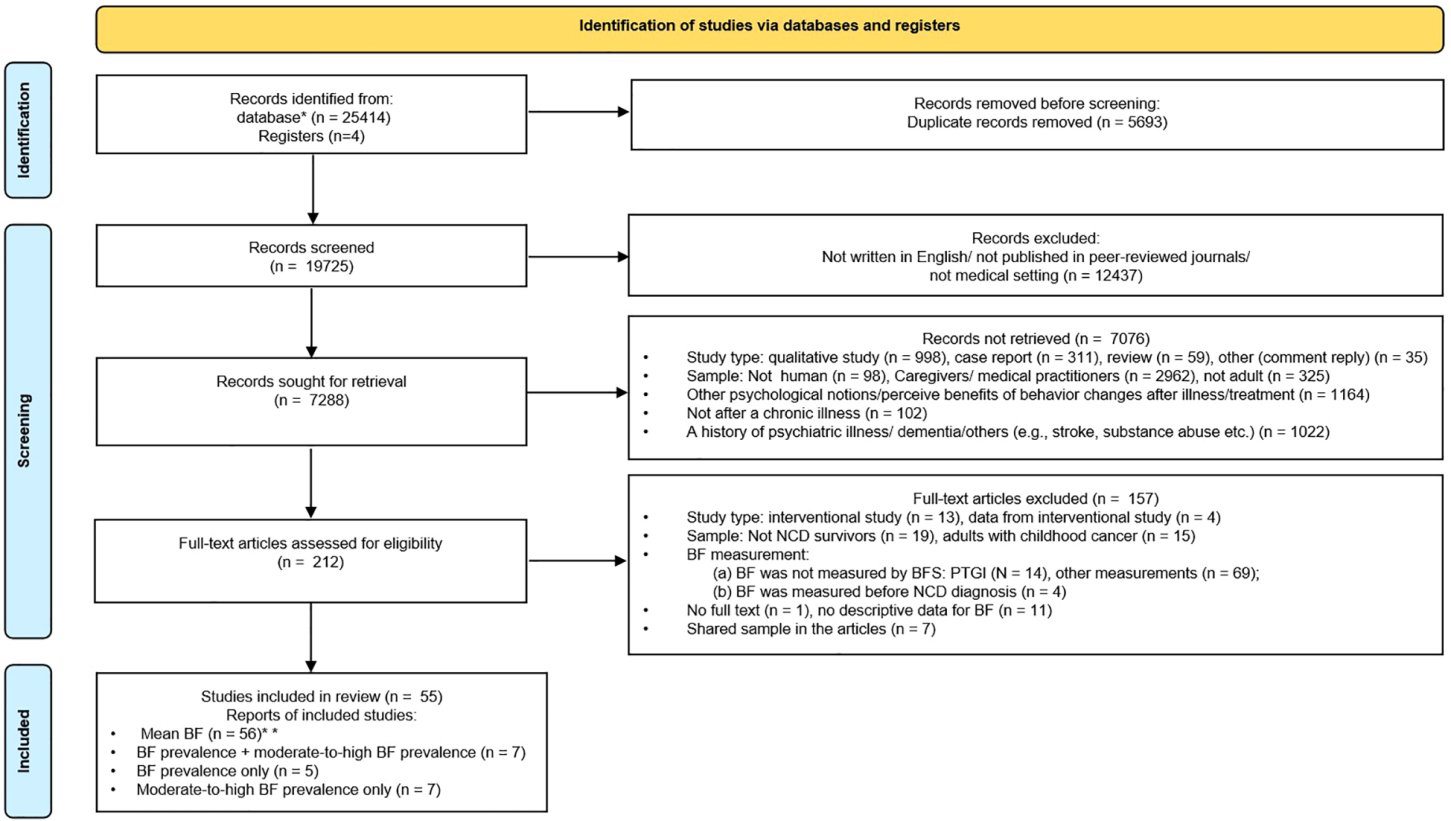
PRISMA flow diagram for this systematic review. *PubMed (Medline) (n = 6,709), Web of Science (WOK, CCC, and SciELO) (n = 7,208), EBSCOhost research databases (PsycINFO, PsycARTICLES, CINAHL, and PSYNDEX literature with PSYNDEX Tests) (n = 1,1497). **There is one study (Weaver et al., 2008) conducted in breast cancer survivors and prostate cancer survivors that reported mean BF according to cancer type. PRISMA, Preferred Reporting Items for Systematic Reviews and Meta-Analyses; BF, benefit finding.

There were 25,414 articles from the electronic databases and an additional four articles from other sources identified through initial searches. After removing duplicates and screening the titles and abstracts of the records, 212 full-text articles were assessed for eligibility (**Figure 1**). Finally, 55 articles (12–15, 17–68) were included in this review, of which 19 articles (12–15, 17–31) reporting the prevalence of BF and 55 articles reporting the mean level of BF were included in the heterogeneity analyses.

### Characteristics of included studies

The general characteristics of 55 included studies are presented in **Table 1**, and detailed studies that reported mean BF are shown in **Supplementary Table 5**. Studies were mainly single-centered, were cross-sectional in design, and focused on the cancer population with sample sizes ≤500. Detailed studies that reported data for BF prevalence are summarized in **Table 2**. Of 19 studies, 11 reported BF prevalence, and 14 reported moderate-to-high BF prevalence.

**Table 1:** Summary of characteristics of all included studies (n = 55) and the studies included in effect size calculation for the prevalence of benefit finding (n = 12) and moderate-to-high benefit finding (n = 14).

Factor	Category	No. studies		
		For mean (n = 55)	For prevalence (n = 12)	For moderate-to-high prevalence (n = 14)
Report year	2000s	18	6	3
	2010s	27	4	8
	2020s	10	2	3
Study quality				
Study design ^0^	Longitudinal	20	3	2
	Cross-sectional	35	9	12
Sample frame	Single-centered	38	11	10
	Population-based	13	1	3
	Cannot be determined	4	0	1
Methodological quality	Strong	2	0	0
	Moderate	18	6	3
	Weak	35	6	11
Sample characteristics				
Diagnosis	**Cancer**	**43**	**6**	**9**
	Various cancers	15	3	3
	Breast cancer	12	1	1
	Head and neck cancer	3	1	1
	Colorectal cancer	4	1	2
	gynecological cancer	3	0	0
	Prostate cancer	3	0	1
	testicular cancer	1	0	0
	Lung cancer	1	0	0
	Brain tumor	1	0	1
	**Non-cancer**	**12**	**6**	**5**
	HIV	3	1	1
	Parkinson’s disease	2	0	1
	Multiple sclerosis	2	2	1
	Rheumatoid arthritis	1	0	1
	Cardiac patients	1	0	0
	Other	3	3	1
Time since diagnosis ^1^	Not given	19	4	5
	<1 year	9	2*	1*
	1–5 years	16	1*	2*
	>5 years	11	5	6
Gender	Female	14	1	0
	Male	4	0	1
	Both	37	11	13
Mean age at survey ^1^	Not given	3	0	1
	≤50	18	6	3
	51–60	18	3	6
	61–70	12	2	2
	≥70	4	1	2
Geographic origin	North America ^3^	22	4	3
	Asia ^4^	12	3	4
	Australia	8	2	2
	Europe ^5^	13	3	5
Sample size ^1,2^	<100	9	2	2
	100–300	21	2	5
	301–500	16	5	4
	>500	9	3	3

Population-based: data from local/regional/national registry. Single-centered: hospital/clinic/medical center/community/hospital registry/university cancer registry.

BF, benefit finding.

^0^Study design for BF analysis.

^1^These categories were arbitrarily determined.

^2^Sample size means those who filled BF measurements in the study.

^3^USA and Canada.

^4^China and Japan.

^5^Germany, Spain, and UK.

*In the subgroup analysis, they were grouped into “≤ 5 years” group.

**Table 2:** Detailed characteristics of the studies included in effect size calculation for the prevalence of benefit finding (n = 19).

No.	First author (year)	Country	n (female %)	Diagnosis	Time since diagnosis M ± SD (range)	Age at Survey M ± SD (range)	BFS	BF prevalence with definition (n = 11)		Moderate-to-high BF prevalence with definition (n = 14)		Study design	
								Definition a	Definition b	Definition c/d	Definition e		
1	Harrington (2008) (17)	UK	76 (51%)	Head and neck cancer	NR	66.9 ± 12.6 (32–97)	17-item ^a^	/	93%	/	NR	C	
2	Jansen (2011) (13)	Germany	483 (38%), N = 470 who filled in BFS	Colorectal cancer	5.4 ± 0.4 (4.8–6.4) years	71.3 ± 9.2	10-item ^b^	98.9%	/	64%	/	C	
3	Kangas (2011) (15)	Australia	70 (77%)	Benign meningioma brain tumors	52.5 months	57.19 ± 11.92 (36–87)	17-item ^b^	NR	/	63%	/	C	
4	Kortte (2010) (19)	USA	87 (32.2%)	Spinal cord injury	NR	47.28 ± 18.5 (18–85)	17-item ^b^	100%*	/	NR	/	C	
5	Kritikos (2021) (20)	USA	326 (29.4%)	Spina bifida	NR	23.44 ± 2.93 (18–30)	17-item ^b^	100%*	/	NR	/	C	
6	Li (2018) (21)	China	772 (47.5%)	Mixed cancer	12.9 ± 13.8 (3–192) months	55.1 ± 12.7 (18–88)	17-item ^b^	100%*	/	76%	/	C
7	Littlewood (2008) (22)	USA	221 (44%)	HIV	7 years since testing positive	40 (22–59)	17-item ^b^	100%^4^	/	72.4% ^d^	/	C	
8	Liu-1 (2018) (23)	China	351 (47.6%)	Mixed cancer	18.94 ± 16.88 weeks	57.34 ± 9.05	22-item ^b^	100% ^4^	/	92.8%*	/	C	
9	Liu-2 (2020) (14)	Germany	6,952 (44.69%), n = 6,831 who filled in BFS	Mixed cancer	8.0 ± 2.2 years	69.1 ± 8.9	10-item ^b^	98.48% ^1^	/	66.02%	/	C	
10	Llewellyn (2013) (24)	UK	103 (29%) N = 100	Head and neck cancer	NR	NR	17-item ^a^	/	NR	/	58.82%*	L	
11	Navarta-Sánchez (2016) (25)	Spain	91 (42.9%)	Parkinson’s disease	102 ± 9.4 (1–50) years	71.9 ± 9.5 (38–93)	17-item ^b^	NR	/	NR	30.4%*	C	
12	Pakenham-1 (2005) (26)	Australia	477 (77%) N = 398 who filled in BFS	Multiple sclerosis	117.24 ± 98.24 (3–624) months	47.77 ± 11.48	19-item ^a^	/	77.4%	/	NR	L	
13	Pakenham-2 (2009) (27)	Australia	388 (82%)	Multiple sclerosis	10.56 ± 8.32 (1 month to 41 years)	49.33 ± 11.31 (21–80)	43-item ^c^	100%	/	55.2% (average score > 2)	/	L	
14	Ramanathan-Elion (2016) (28)	USA	206 (44.7%), among them 142 were NCDs	Spinal cord dysfunction, ischemic, or hemorrhagic stroke	NR	Total sample: 57.27 ± 17.49 (18–92) N = 142: 52.77 ± 18.88 (18–91)	17-item ^b^	100% ^d^	/	71.9% ^a^	/	C	
15	Sato (2008) (12)	Japan	364 (79.1%)	Rheumatoid arthritis	10.6 ± 7.4 years	45.5 ± 8.4	11-item ^a^	/	NR	/	97.5%	C	
16	Wang-2 (2015a) (30)	China	658 (100%)	Breast cancer	At least 4 weeks	47.52 ± 8.23 (25–70)	17-item ^b^	100%*	NR	/	NR	L	
17	Wen (2016) (31)	China	148 (100%)	Breast cancer	NR	54.1 ± 7.39	6-item ^a^	/	NR	/	88.5%*	C	
18	Jahnen (2023) (18)	Germany	2,298 (0)	Prostate cancer	NR	69.5 ± 18.2	17-item ^b^	NR	/	49.6%	/	C	
19	Sheikh-Wu (2023) (29)	USA	117 (51%)	Colorectal cancer	NR	55.31 ± 11.62 (21–88)	17-item ^b^	NR	/	88.03%	/	C	

Definition a: the percentage of participants whose mean item score >1. Definition b: the percentage of participants whose mean item score >2 or ≥3. Definition c: the percentage of participants whose mean item score >2 or ≥3. Definition d: the percentage of participants whose mean item score >2. Definition e: the percentage of participants whose mean item score ≥4.

n, number of samples to measure BF; M, mean; SD, standard deviation; BF, benefit finding; NR, not reported; C, cross-sectional; L, longitudinal; NCDs, non-communicable chronic diseases.

*Inferred from the range of the total BFS scores; minimal BFS/number of items >1 indicated that 100% of participants experienced at least one benefit.

a Likert-type scale from 1 (strongly disagree) to 5 (strongly agree).

b Likert-type scale from 1 (not at all) to 5 (extremely).

c Likert-type scale from 1 (not at all) to 3 (great deal).

d Calculated from the original data provided by the authors of the included studies.

### Quality assessment of included studies

Of the 55 included studies, 35 received a “weak” rating, 18 received a “moderate” rating, and only two were rated “strong” in quality assessment (**Supplementary Figure 1**). Most articles scored poorly on the aspects of “study design”. Thirty-one articles that rated “weak” in study design had a cross-sectional design. For “confounders”, 13 studies received the highest ratings. This was due to the changes applied to the rating tool (**Supplementary Table 2**), which led to a more liberal rating. For BF means, 51 studies reported crude BF means, six reported BF means adjusted for confounders (numbers do not add up to 55, as two studies (19, 22) provided original data at the request of Author 1). For BF prevalence, one study reported prevalence adjusted for confounders, and 18 reported crude prevalence (authors of seven studies provided additional results on the prevalence beyond what was published, at the request of Author 1).

### Heterogeneity test and combination of BF mean level and BF prevalence

As shown in **Table 3**, in 10,720 participants from 12 included studies, the pooled BF prevalence was 97.5% (95% CI: 93.9%–100%) with high heterogeneity (τ^2^ = 0.004, I^2^ = 94.1%, *p* < 0.01). The pooled moderate-to-high BF prevalence from 12,363 observations of 14 included studies was 74.1% with high heterogeneity (τ^2^ = 1.186, I^2^ = 97.7%, *p* < 0.01). Stratified by cancer and non-cancer samples, the BF prevalence (cancer, 99.4%; non-cancer, 96.1%, *χ*^2^ = 158.58, *p* < 0.001) and moderate-to-high (M-H) BF prevalence (cancer, 74.8%; non-cancer, 72.9%, *χ*^2^ = 21.54, *p* < 0.001) were statistically different. The heterogeneity remained high. A total of 25,972 observations from 56 estimates that were reported in the 55 included studies were included in the mean BF analysis. The pooled mean BF level was 3.25 with a 95% CI of 3.11 to 3.40. However, the between-study heterogeneity was high (τ^2^ = 0.294, I^2^ = 99.5%, *p* < 0.01).

**Table 3:** Heterogeneity test and combination of effect sizes.

Item	Sample	Heterogeneity test				Combination of effect sizes					Cancer vs. non-cancer
		Q	P_Q_	I^2^	τ^2^	Transformation	ES	n	N	95% CI	
BF prevalence	SNCD	186.52	<0.0001	94.1%	0.004	Log transformation	97.5%	12	10,720	93.9%–100%	
	Cancer	94.81	<0.0001	94.7%	<0.0001	Log transformation	99.4%	6	9,158	98.8%–100%	*χ*^2^ = 158.58
	Non-cancer	89.33	<0.0001	94.4%	0.010	Log transformation	96.1%	6	1,562	88.7%–100%	*p* < 0.001
Moderate-to-high BF prevalence	SNCD	555.77	<0.0001	97.7%	1.186	Logit transformation	74.1%	14	12,363	61.6%–83.6%	
	Cancer	505.79	<0.0001	98.1%	0.683	Logit transformation	74.8%	9	11,157	63.0%–83.7%	*χ*^2^ = 21.54
	Non-cancer	287.73	<0.0001	97.2%	2.143	Logit transformation	72.9%	5	1,206	42.3%–98.9%	*p* < 0.001
BF mean level	SNCD	11,557.69	<0.0001	99.5%	0.294	Untransformed (raw) mean	3.254	55	25,972	3.111–3.397	
	Cancer	9,805.14	<0.0001	99.6%	0.303	Untransformed (raw) mean	3.272	43	23,645	3.108–3.436	*T* = 0.467
	Non-cancer	1,700.15	<0.0001	99.4%	0.278	Untransformed (raw) mean	3.191	12	2,327	2.889–3.492	*p* = 0.642

Random-effects model was used in each test.

BF, benefit finding; SNCD, somatic non-communicable chronic disease; ES, overall effect size; n, number of primary studies; N, total sample size; CI, confidence interval.

### Subgroup analyses

Subgroup analysis results are presented in **Supplementary Table 6**. Considering the significant difference between cancer and non-cancer populations, subgroup analyses stratified by cancer and non-cancer were conducted to determine factors associated with BF prevalence (**Table 4**) and moderate-to-high BF prevalence (**Table 5**).

**Table 4:** Subgroup analysis results 1-BF prevalence in cancer and non-cancer populations.

Variable		Cancer (n = 6)					Non-cancer (n = 6)				
		No. studies	No. survivors	Mean (95% CI)*	I^2^ (%)	Differences between subgroups (*p*)*	No. studies	No. survivors	Mean (95% CI)*	I^2^ (%)	Differences between subgroups (*p*)*
Report year	2000s	1	76	0.93 (0.85, 0.98)	–	*χ*^2^ = 59.86, ***p* < 0.01**	3	1,007	0.92 (0.78, 1.00)	98	*χ*^2^ = 0.98, *p* = 0.61
	2010s	4	2,251	1.00 (1.00, 1.00)	39		2	229	1.00 (0.99, 1.00)	0	
	2020–2023	1	6,831	0.98 (0.98, 0.99)	–		1	326	1.00 (0.99, 1.00)	–	
Study design	Longitudinal	1	658	1.00 (0.99, 1.00)	–	*χ*^2^ = 2.88, *p* = 0.09	2	786	0.88 (0.69, 1.00)	99	*χ*^2^ = 0.98, *p* = 0.32
	Cross-sectional	5	8,500	0.99 (0.98, 1.00)	95		4	776	1.00 (1.00, 1.00)	0	
Sample frame	Single-centered	5	2,327	1.00 (1.00, 1.00)	59	*χ*^2^ = 43.64, ***p* < 0.01**	6	1,562	0.96 (0.89, 1.00)	94	*-*
	Population-based	1	6,831	0.98 (0.98, 0.99)	–		–	–	–	–	
Methodological quality	Moderate	1	470	0.99 (0.98, 1.00)	–	*χ*^2^ = 0.87, *p* = 0.35	5	1,420	0.95 (0.86, 1.00)	96	*χ*^2^ = 0.97, *p* = 0.33
	Weak	5	8,688	1.00 (0.99, 1.00)	96		1	142	1.00 (0.97, 1.00)	–	
Time since diagnosis ^1^	<5 years	3#	1,781	1.00 (1.00, 1.00)	0	*χ*^2^ = 93.95, ***p* < 0.01**	–	–	–	–	*χ*^2^ = 0.98, *p* = 0.32
	>5 years	2##	7,301	0.98 (0.98, 0.99)	0		3	1,007	0.92 (0.78, 1.00)	98	
	Not given	1###	76	0.93 (0.85, 0.98)	–		3	555	1.00 (1.00, 1.00)	0	
Gender	Female	1	658	1.00 (0.99, 1.00)	–	*χ*^2^ = 2.88, *p* = 0.09	–	–	–	–	*-*
	Male	–	–	–	–		–	–	–	–	
	Both (ref.)	5	8,500	0.99 (0.98, 1.00)	95		6	1,562	0.96 (0.89, 1.00)	94	
Mean age at survey ^1^	q 50	1	658	1.00 (0.99, 1.00)	–	*χ*^2^ = 46.04, ***p* < 0.01**	5	1,420	0.95 (0.86, 1.00)	96	*χ*^2^ = 0.97, *p* = 0.33
	51–60	2	1,123	1.00 (1.00, 1.00)	0		1	142	1.00 (0.97, 1.00)	–	
	>60	3	7,377	0.99 (0.98, 0.99)	49		–	–	–	–	
Geographic origin	North America	–	–	–	–	*χ*^2^ = 46.04, ***p* < 0.01**	4**	776	1.00 (1.00, 1.00)	0	*χ*^2^ = 0.98, *p* = 0.32
	Asia ^4^	3	1,781	1.00 (1.00, 1.00)	0		–	–	–	–	
	Australia	–	–	–	–		2***	786	0.88 (0.69, 1.00)	99	
	Europe	3	7,377	0.99 (0.98, 0.99)	49		–	–	–	–	
Sample size ^1,2^	<100	1	76	0.93 (0.85, 0.98)	–	*χ*^2^ = 4.27, *p* = 0.12	1	87	1.00 (0.96, 1.00)	–	*χ*^2^ = 0.98, *p* = 0.61
	100–300	–	–	–	–		2	363	1.00 (0.99, 1.00)	0	
	301–500	2	821	1.00 (0.99, 1.00)	76		3	1,112	0.92 (0.78, 1.00)	98	
	>500	3	8,261	0.99 (0.99, 1.00)	98		–	–	–	–	

Population-based: data from local/regional/national registry. Single-centered: hospital/clinic/medical center/community/hospital registry/university cancer registry.

CI, confidence interval; BF, benefit finding.

*Random-effects model.

^1^These categories were arbitrarily determined.

^2^Sample size means those who filled BF measurements in the study.

^#^Studies of Nos. 6, 8, and 16 in **Table 2** from China.

^##^Studies of No. 2 (colorectal cancer) and No. 9 (mixed cancer sample: breast, colorectal, and prostate cancers) in **Table 2**; all samples came from Germany.

^###^Studies of No. 1 in **Table 2**, head and neck cancer sample.

^**^Studies of Nos. 4, 5, 7 (HIV sample), and 14 in **Table 2**.

^***^Studies of Nos. 12 and 13 in **Table 2**; multiple sclerosis from Australia. Bold values are p < 0.05.

**Table 5:** Subgroup analysis results 2-moderate-to-high BF prevalence in cancer and non-cancer populations.

Variable		Cancer (n = 9)					Non-cancer (n = 5)				
		No. studies	No. survivors	Mean (95% CI)*	I^2^ (%)	Differences between subgroups (*p*)*	No. studies	No. survivors	Mean (95% CI)*	I^2^ (%)	Differences between subgroups (*p*)*
Report year	2000s	–	–	–	–	*χ*^2^ = 0.38, *p* = 0.54	3	973	0.83 (0.48, 0.96)	98	*χ*^2^ = 2.08, *p* = 0.15
	2010s	6	1,911	0.77 (0.63, 0.87)	95		2	233	0.52 (0.24, 0.78)	97	
	2020–2023	3	9,246	0.70 (0.48, 0.86)	–		–	–	–	–	
Study design	Longitudinal	1	100	0.59 (0.49, 0.69)	–	*χ*^2^ = 4.93, ***p* = 0.03**	1	388	0.55 (0.50, 0.60)	–	*χ*^2^ = 1.47, *p* = 0.22
	Cross-sectional	8	11,057	0.76 (0.64, 0.85)	98		4	818	0.76 (0.40, 0.94)	98	
Sample frame	Single-centered	6	4,139	0.75 (0.59, 0.86)	98	*χ*^2^ = 2.90, *p* = 0.23	4	1115	0.81 (0.54, 0.94)	97	*χ*^2^ = 10.43, ***p* < 0.01**
	Population-based	2	6,948	0.78 (0.59, 0.90)	95		1	91	0.31 (0.22, 0.41)	–	
	Cannot be determined	1	70	0.63 (0.50, 0.74)	–		–	–	–	–	
Methodological quality	Moderate	1	470	0.64 (0.59, 0.68)	–	*χ*^2^ = 3.24, *p* = 0.07	2	609	0.64 (0.51, 0.75)	94	*χ*^2^ = 0.40, *p* = 0.53
	Weak	8	10,687	0.76 (0.63, 0.85)	98		3	597	0.78 (0.30, 0.97)	98	
Time since diagnosis ^1^	<5 years	3	1,193	0.81 (0.62, 0.92)	96	*χ*^2^ = 3.27, *p* = 0.19	–	–	–	–	*χ*^2^ = 0.01, *p* = 0.94
	>5 years	2	7,301	0.66 (0.65, 0.67)	0		4	1,064	0.73 (0.35, 0.93)	98	
	Not given	4	2,663	0.75 (0.54, 0.88)	97		1	142	0.72 (0.64, 0.79)	–	
Gender	Male	1	2,298	0.50 (0.48, 0.52)	–	*χ*^2^ = 19.37, ***p* < 0.01**	–	–	–	–	–
	Both	8	8,859	0.77 (0.66, 0.86)	96		5	1,206	0.62 (0.43, 0.89)	98	
Mean age at survey ^1^	q 50	–	–	–	–	*χ*^2^ = 13.18, ***p* < 0.01**	3	973	0.83 (0.48, 0.96)	98	*χ*^2^ = 37.80, ***p* < 0.01**
	51–60	5	1,458	0.84 (0.74, 0.91)	94		1	91	0.31 (0.22, 0.41)	–	
	>60	3	9,599	0.60 (0.51, 0.68)	99		1	142	0.72 (0.64, 0.79)	–	
	Not given	1	100	0.59 (0.49, 0.69)	–		–	–	–	–	
Geographic origin	North America	1	117	0.88 (0.81, 0.93)	–	*χ*^2^ = 36.32, ***p* < 0.01**	2	363	0.72 (0.67, 0.77)	0	*χ*^2^ = 144.73, ***p* < 0.01**
	Asia	3	1,271	0.87 (0.77, 0.93)	96		1	364	0.97 (0.95, 0.98)	–	
	Australia	1	70	0.63 (0.50, 0.74)	–		1	388	0.55 (0.50, 0.60)	–	
	Europe	4	9,699	0.60 (0.53, 0.66)	98		1	91	0.31 (0.22, 0.41)	–	
Sample size ^1,2^	<100	1	70	0.63 (0.50, 0.74)	–	*χ*^2^ = 4.80, *p* = 0.19	1	91	0.31 (0.22, 0.41)	–	*χ*^2^ = 48.84, ***p* < 0.01**
	100–300	3	365	0.81 (0.64, 0.91)	94		2	363	0.72 (0.67, 0.77)	0	
	301–500	2	821	0.83 (0.54, 0.95)	99		2	752	0.87 (0.38, 0.99)	99	
	>500	3	9,901	0.64 (0.51, 0.76)	99		–	–	–	–	
Diagnosis	Mixed cancer	3	7,954	0.81 (0.63, 0.91)	98	*χ*^2^ = 82.48, ***p* < 0.01**	Diagnosis categories details for non-cancer; please refer to **Table 1**.				
	Colorectal cancer	2	587	0.78 (0.56, 0.90)	96						
	Breast cancer	1	148	0.89 (0.82, 0.93)	–						
	Prostate cancer	1	2,298	0.50 (0.48, 0.52)	–						
	Head and neck cancer	1	100	0.59 (0.49, 0.69)	–						
	Brain tumor	1	70	0.63 (0.50, 0.74)	–						

Population-based: data from local/regional/national registry. Single-centered: hospital/clinic/medical center/community/hospital registry/university cancer registry.

CI, confidence interval; BF, benefit finding.

*Random-effects model.

^1^These categories were arbitrarily determined.

^2^Sample size means those who filled BF measurements in the study. Bold values are p < 0.05.

BF prevalence (**Table 4**): For the cancer group (n = 6 studies), the tests for subgroup differences were statistically significant for report year, sample frame, time since diagnosis, mean age at survey, and geographic origin. In three studies on Chinese cancer survivors who had a time since diagnosis of <5 years and were <60 years old during the survey, the pooled BF prevalence was 100%. In the other three European studies conducted in samples with a mean age of >60 years, the pooled BF prevalence was 99%. For the non-cancer group, no statistically significant differences were found.

Moderate-to-high BF prevalence (**Table 5**): For the cancer group, the tests for subgroup differences were statistically significant for study design, gender, mean age at survey, geographic origin, and type of cancer for moderate-to-high BF prevalence. Moderate-to-high BF prevalence was the highest among breast cancer survivors (89%, 95% CI: 82%–93%), and prostate cancer survivors had the lowest prevalence (50%, 95% CI: 48%–52%) (**Table 5**). The moderate-to-high BF prevalence was the lowest in cancer survivors with a mean age of 60+ years. For the non-cancer group, the five included studies reported on samples with different diagnoses (**Table 2**); the tests for subgroup differences were statistically significant for sample frame, geographic origin, and sample size for moderate-to-high BF prevalence. However, heterogeneity remained very high within the subgroup (I^2^ > 90%).

### Meta-regression

The meta-regression results on pooled BF means showed that report study design, methodological quality, time since diagnosis, gender, and mean age at survey may potentially contribute to the high heterogeneity of the combined BF mean level; see **Table 6**. The overall proportion of variance explained by these covariates in the final model was 43.53% (R^2^ = 0.4353, F = 64.31, *p* < 0.001). Subgroup analyses were conducted subsequently on the factors identified in the meta-regression. Only study design (*χ*^2^ = 6.56, *p* = 0.01) and mean age at survey (*χ*^2^ = 23.11, *p* < 0.01) showed a significant difference in mean BF scores between the subgroups (**Supplementary Table 5**). The BF mean level in studies with a longitudinal study design (3.48, 95% CI: 3.29–3.66) was higher than that in cross-sectional (3.13, 95% CI: 2.94–3.32) studies. The mean BF in the group with a mean age of 70+ years (2.57, 95% CI: 2.01–3.14) was significantly lower than the groups with a mean age of 51–60 years (3.58, 95% CI: 3.43–3.74) and 61–70 years (3.44, 95% CI: 3.29–3.58).

**Table 6:** The meta-regression results for BF mean level (number of studies = 55).

Variable		Coefficient	Standard error	95% CI	
				LL	UL
Report year	2000s (ref.)	–	–	–	–
	2010s	0.1684	0.1775	−0.1796	0.5163
	2020–2023	0.2488	0.2389	−0.2193	0.7170
Study design	Longitudinal	0.4693**	0.1654	0.1451	0.7936
	Cross-sectional (ref.)	–	–	–	–
Sample frame	Single-centered (ref.)	–	–	–	–
	Population-based	−0.2154	0.2073	−0.6216	0.1908
	Cannot be determined	−0.6913	0.4154	−1.5053	0.1228
Methodological quality	Strong	−0.1079	0.3631	−0.8195	0.6037
	Moderate (ref.)	–	–	–	–
	Weak	0.3599*	0.1813	0.0045	0.7153
Diagnosis	Cancer	−0.2523	0.2441	−0.7307	0.2262
	Non-cancer (ref.)	**-**	**-**	**-**	**-**
Time since diagnosis ^1^	<1 year (ref.)	–	–	–	–
	1–5 years	0.0928	0.2350	−0.3679	0.5534
	>5 years	0.5192*	0.2403	0.0483	0.9901
	Not given	0.0209	0.1990	−0.3691	0.4109
Gender	Female	−0.4502*	0.2245	−0.8903	−0.0102
	Male	−0.1562	0.2588	−0.6634	0.3510
	Both (ref.)	–	–	–	–
Mean age at survey ^1^	q 50 (ref.)	–	–	–	–
	51–60	0.4682*	0.1889	0.0980	0.8385
	61–70	0.2937	0.3013	−0.2968	0.8842
	>70	−0.6541*	0.3337	−1.3080	−0.0001
	Not given	−0.4100	0.3422	−1.0807	0.2606
Geographic origin	North America ^3^ (ref.)	–	–	–	–
	Asia ^4^	−0.2319	0.2070	−0.6376	0.1737
	Australia	−0.2535	0.2452	−0.7341	0.2271
	Europe ^5^	−0.2604	0.2250	−0.7014	0.1806
Sample size ^1,2^	<100 (ref.)	–	–	–	–
	100–300	−0.0266	0.1922	−0.4033	0.3502
	301–500	0.1340	0.2329	−0.3226	0.5905
	>500	−0.3946	0.2814	−0.9462	0.1569

Mixed-effects model, R^2^ (amount of heterogeneity accounted for) = 43.53%. Population-based: data from local/regional/national registry. Single-centered: hospital/clinic/medical center/community/hospital registry/university cancer registry.

CI, confidence interval; LL, lower limit; UL, upper limit; ref., reference group; BF, benefit finding.

^0^Study design for BF analysis.

^1^These categories were arbitrarily determined.

^2^Sample size means those who filled BF measurements in the study.

^3^USA and Canada.

^4^China and Japan.

^5^Germany, Spain, and the UK.

*p < 0.05.

**p < 0.01.

### Sensitivity analyses and publication bias

In sensitivity analyses, the pooled BF mean, the pooled BF prevalence, and the pooled moderate-to-high BF prevalence (**Supplementary Figures 2A–C**) did not significantly change when omitting any of the studies. The cluster-robust variant of Egger’s test (linear regression test of funnel plot asymmetry) did not detect any publication bias (*p* = 0.15 for BF prevalence, *p* = 0.22 for moderate-to-high BF prevalence, and *p* = 0.46 for means). However, the funnel plots seemed asymmetric (**Supplementary Figure 3**), which may be due to survivor bias and the inherent heterogeneity among the included studies.

## Discussion

To the best of our knowledge, this is the first systematic review of meta-analyses of BF prevalence and mean, showing that almost all pSNCDs reported BF, with 74% reporting moderate-to-high BF. This review revealed that age at survey is associated with the BF mean and BF prevalence of pSNCDs. Specifically, pSNCDs aged 60 years or older were less likely to report moderate-to-high BF compared with those aged <60 years. In particular, those older than 70 years tend to report the lowest levels of BF. Although we observed an association between geographic origin and BF prevalence, the interaction between geographic origin and mean age at survey must also be considered.

Age is an influencing factor in BF levels in pSNCDs. In this review, older pSNCDs (60 years or older) were less likely to report BF. The results suggest that multiple physical, psychological, and social changes after the diagnosis of SNCDs may be less challenging for older persons, thereby reducing the potential for the initiation of the BF process. Older adults may have more experience with ultimately unalterable life cycle events (e.g., bereavement and retirement) compared with young adults and thus may be able to endure greater numbers and severity of challenging events than younger adults (14). However, older adults also experience more happiness in life cycle events (e.g., being a grandparent) that could remind them of their current level of well-being. When facing challenging and stressful events, including SNCDs, older adults may already have the capacity and more choices to focus on the positive (69). One researcher suggested that older adults are more likely to have a perceived sense of reaching inwardly (the self) and outwardly (with others) (70), which may help them take on a broader perspective in the face of challenges (71). They are less likely to have an exaggerated discrepancy between their original beliefs (toward the world, self, and self-in-world) and the meaning of the challenge they are facing. Older persons may have a more stable state and do not feel as much stress as younger adults do when facing SNCDs. Therefore, as less distress would be perceived, less positive changes (BF or posttraumatic growth (14), related but distinct concept (13)) would be experienced (72). However, only a few of the included studies reported age-adjusted BF prevalence and mean or reported prevalence and means stratified by age groups. Future studies are highly recommended to take age into consideration when reporting BF in pSNCDs. As very few interviews have been performed to explore BF in-depth, qualitative studies are also needed to explore whether there could be other negative or challenging events that older adults have experienced, which render SNCDs less of a challenge as compared to other life-threatening events to trigger BF for pSNCDs.

All studies included in this review used the BFS to measure BF with a Likert-type response ordinal scale. However, the semantic scale and the number of response choices may differ in the included studies. Two semantic scales were employed: unipolar response (not at all–very much) or bipolar response (strongly disagree–strongly agree). This review employed the original unipolar response for two practical reasons: the original BFS was designed with this response scale; the bipolar response could be transformed to a unipolar response, which allowed more data for pooled prevalence and mean analysis. A previous study compared the two semantic scales and found that the unipolar response outperforms the bipolar response for non-socially desirable positive valence questions (73). Bipolar response scales are prone to acquiescence bias, in that respondents would be more likely to agree than disagree, especially in positive valence questions (74). Normally, the bipolar response scale has a zero point in the middle, which can be either explicit (neither agree nor disagree) or implicit (change from agree to disagree) (73). In our review, the bipolar response scale included studies using a 1–5 scoring point, which made 3 points in the middle, while the unipolar response scale indicated a moderate degree of 3 points. Our transformation and definition greatly reduced the error between the two scoring methods in the statistical analysis. However, the problem of item reliability demonstrated by the bipolar response scale has not been eliminated because respondents still cannot indicate the direction, intensity, and neutrality when scoring 3 in the 5-point Likert scale ranging from strongly disagree to strongly agree. Our review suggests that future studies could adhere to the original unipolar response scale scoring method while applying or cross-culturally translating the BFS.

Furthermore, we also found that BF prevalence varies depending on the cut-off used. The pooled BF prevalence was 97.5% when the cut-off score = 1 was used. If raising the cut-off score to 3, the moderate-to-high BF prevalence was 74.1%. The cut-off score of 3 is comparable to the pooled mean BF level of 3.25. A recent study (29) found that in moderate-to-high BF (mean score ≥3.24), BF moderates the relationship between symptom characteristics (e.g., anal pain) and quality of life. As more included studies reported moderate-to-high BF prevalence, this systematic review recommends reporting moderate-to-high BF prevalence in future studies.

This systematic review attempted to synthesize a pooled BF prevalence and BF mean level in pSNCDs using a rigorous methodology based on international reporting guidelines. However, some limitations should be noted. We only used the BFS, developed first for use in cancer populations, which explains that most included studies are on cancer samples. The prevalence rates of BF in cancer and non-cancer samples differed. Therefore, the results of this study may not be directly generalizable to all pSNCDs. BF prevalence is suggested to be reported stratified by disease in future studies. High heterogeneity in the pooled results could be related to the methodology of the included studies (e.g., Likert-type scaling in the BFS used) and demographic/clinical characteristics not mentioned in most studies (e.g., marital status). Factors such as gender and disease status were not analyzed in detail; e.g., majority of the included studies are gender-specific (e.g., focused on breast cancer, testicular cancer, or men with HIV).

The results of our systematic review have implications for clinical practice. Research suggests that moderate-to-high BF aids in symptom management and improves quality of life (29). To identify potentially “at risk” pSNCDs (i.e., those who report none-to-low BF), it is recommended that clinical practitioners and researchers screen for BF using the BFS with the original unipolar rating scale and a cut-off suggested in this review, in particular, for older pSNCDs (60 years or older) who were less likely to report BF. However, pSNCDs with moderate-to-high BF reported lower quality of life than those with none-to-low BF (75). Therefore, studies investigating and comparing the clinical results of BF (none-to-low vs. moderate-to-high) are still needed to support that pSNCDs with none-to-low BF are really at risk, e.g., qualitative studies, survival analysis. The necessity for interventions aimed at improving BF needs further validation. The high heterogeneity of the pooled BF outcome and poor quality of the included studies in this review suggest the need for high-quality studies on BF outcomes stratified by factors, e.g., time since diagnosis, mean age at diagnosis, and geographic origin. It is recommended that future studies investigating BF in pSNCDs should control for relevant confounders.

## Conclusions

The pSNCDs generally reported a moderate level of BF, with a 74% moderate-to-high BF prevalence and a 3.32 BF mean. BF prevalence and BF mean level were associated with age at survey. Future studies of high quality, both qualitative and quantitative, are needed to fully understand how SNCD survivors find/perceive benefits, especially for older adults aged 60 years and above. These findings may be used to inform the development of patient-centered interventions and thereby assist in improving health outcomes and relieving the negative effects of SNCDs.

## Data Availability

The original contributions presented in the study are included in the article/**Supplementary Material**. Further inquiries can be directed to the corresponding author.
