# Supplementary material for: Benefit finding in adults living with somatic non-communicable chronic disease: a systematic review on the mean level and the prevalence
Source: Front Psychiatry. 2026 Jan 26;16:1452218. doi: 10.3389/fpsyt.2025.1452218 (PMC12883746; doi:10.3389/fpsyt.2025.1452218)

**Supplementary Table 1 Search strategy**

| **PICO-MODEL** | MeSH | Own keywords |  |
| --- | --- | --- | --- |
| Benefit finding | **Quality of life/psychological**  **"Adaptation, Psychological"**  Mental health  Psychological well-being | **„benefit finding*“**  **„finding benefit“**  **„mental well-being“**  **“psychological well-being”**  **„mental health“**  **„perceived benefit“**  **„positive change“**  **„positive growth“**  **„positive adaption“**  **„positive reappraisal“** | (Benefit*[tw] AND (Finding*[tw] OR Perceived[tw]))  ((Well-being[tw] OR health[tw]) AND (mental[tw] OR psychological[tw]))  (Positive[tw] AND (change*[tw] OR growth[tw] OR adaption*[tw] OR reappraisal*[tw]))  “psychological adaption”[tw]  "Adaptation, Psychological"[Mesh]  "Quality of Life/psychology"[Mesh]  "Mental health"[Mesh]  "Psychological well-being"[Mesh] |
| Adults, 18 years and above | **Adult** | **Adult***  **„above 18 years“** | Adult*[tw] OR “18 years and above“[tw] OR adult[mesh] |
| non-communicable chronic illness | **Chronic Disease**  Chronic Disease/Psychology  Noncommunicable Diseases | **Chronic**  Noncommunicable  Non-communicable  Noninfectious  Non-infectious | chronic[tw]  Chronic Disease[mesh] |
| Benefit finding scale | **“Surveys and Questionnaires”[MeSH Terms)**  Statistics and numerical data  (as subheading) | **„Benefit finding scale“**  **„Benefit finding questionnaire*“**  BFS | „benefit finding scale“[tw]  ("benefit find*"[All Fields] AND ("scale*"[All Fields] OR "questionnaire*"[All Fields]))  “Surveys and Questionnaires”[MeSH]  "Surveys and Questionnaires/statistics and numerical data"[Mesh]) |
| Language filter | Chinese, English | | |
| Time span | 1973 - 2021 | | |

**Supplementary Table 2. Modified Quality Assessment Tool**

| **Component Ratings** | **Dictionary** |
| --- | --- |
| **A) SELECTION BIAS** | |
| **(Q1) Are the individuals selected to participate in the study likely to be representative of the target population?**   1. 1 Very likely 2. 2 Somewhat likely 3. 3 Not likely 4. 4 Can’t tell | **(Q1)** Participants are more likely to be representative of the target population if they are randomly selected from a comprehensive list of individuals in the target population (score very likely). They may not be representative if they are referred from a source (e.g. clinic) in a systematic manner (score somewhat likely) or self-referred (score not likely). |
| **(Q2) What percentage of selected individuals agreed to participate?**   1. 1 80 - 100% agreement 2. 2 60 – 79% agreement 3. 3 less than 60% agreement 4. 4 Not applicable 5. 5 Can’t tell | **(Q2)** Refers to the % of subjects in the control and intervention groups that agreed to participate in the study before they were assigned to intervention or control groups. |
| \| **RATE THIS SECTION** \| **STRONG** \| **MODERATE** \| **WEAK** \| \| --- \| --- \| --- \| --- \| \| See dictionary \| 1 \| 2 \| 3 \| | **Strong:** The selected individuals are very likely to be representative of the target population (Q1 is 1) **and** there is greater than 80% participation (Q2 is 1).  **Moderate:** The selected individuals are at least somewhat likely to be representative of the target population (Q1 is 1 or 2); **and** there is 60 - 79% participation (Q2 is 2). ‘Moderate’ may also be assigned if Q1 is 1 or 2 and Q2 is 5 (can’t tell).  **Weak:** The selected individuals are not likely to be representative of the target population (Q1 is 3); **or** there is less than 60% participation (Q2 is 3) **or** selection is not described (Q1 is 4); and the level of participation is not described (Q2 is 5). |
| **B) DESIGN** | |
| **Indicate the study design**   1. 1 Cohort analytic (two group pre + post) 2. 2 Case-control 3. 3 Cohort (one group pre+post (before and after)) 4. 4 Interrupted time series 5. 5 Other specify_________________________ 6. 6 Can’t tell | Cohort analytic (two group pre and post). An observational study design where groups are assembled according to whether or not exposure to the intervention has occurred. Exposure to the intervention is not under the control of the investigators. Study groups might be nonequivalent or not comparable on some feature that affects outcome.  Case control study: A retrospective study design where the investigators gather ‘cases’ of people who already have the outcome of interest and ‘controls’ who do not. Both groups are then questioned or their records examined about whether they received the intervention exposure of interest.  Cohort (one group pre + post (before and after): The same group is pretested, given an intervention, and tested immediately after the intervention. The intervention group, by means of the pretest, act as their own control group.  Interrupted time series: A study that uses observations at multiple time points before and after an intervention (the ‘interruption’). The design attempts to detect whether the intervention has had an effect significantly greater than any underlying trend over time.  Other: One time surveys or interviews |
| \| **RATE THIS SECTION** \| **STRONG** \| **MODERATE** \| **WEAK** \| \| --- \| --- \| --- \| --- \| \| See dictionary \| 1 \| 2 \| 3 \| | **Strong:** will be assigned to those articles that described RCTs and CCTs.  **Moderate:** will be assigned to those that described a cohort analytic study, a case control study, a cohort design, or an interrupted time series.  **Weak:** will be assigned to those that used any other method or did not state the method used. |
| **C) CONFOUNDERS** | |
| **(Q1) Were there key potential confounding variables for the outcome- the prevalence of BF?**   1. 1 Yes 2. 2 No 3. 3 Can’t tell   **The following are examples of main confounders:**   1. 1. Race (if applicable) 2. 2. Sex 3. 3. Marital status/ family 4. 4. Age (older, adult, adolescent, child) 5. 5. SES (income or class) 6. 6. Education 7. 7. Health status (usually in the restriction of the illness type) | By definition, a confounder is a variable that is associated with the exposure and causally related to the outcome of interest. All key factors that may be associated both with the exposure of interest and the outcome–that are not of interest to the research question–should be controlled for in the analyses. The authors should indicate if confounders were controlled in the design or in the analysis.  In non-randomized studies if the effect of the main confounders was not investigated or no adjustment was made in the final analyses the question should be answered as not controlled.  If one confounder was mentioned in the sampling restriction or in the matching process (matching groups by the factor we considered as the confounder), then considered as controlled confounder.  If the confounder was measured in the study, but no significant difference between the groups shown then considered as controlled.  If one confounder (e.g. age) was used to be the stratification factor, then considered as the controlled confounder.  If adjusted-prevalence was reported, then the paper considered the adjusted factor as the confounder. |
| **(Q2) If yes, indicate the percentage of relevant confounders that were controlled (either in the design (e.g. stratification, matching) or analysis)?**   1. 1 80 – 100% (most) 2. 2 60 – 79% (some) 3. 3 Less than 60% (few or none) 4. 4 Can’t Tell |  |
| \| **RATE THIS SECTION** \| **STRONG** \| **MODERATE** \| **WEAK** \| \| --- \| --- \| --- \| --- \| \| See dictionary \| 1 \| 2 \| 3 \| | **Strong:** will be assigned to those articles that controlled for at least 80% of relevant confounders (Q1 is 2); **or** (Q2 is 1).  **Moderate:** will be given to those studies that controlled for 60 – 79% of relevant confounders (Q1 is 1) **and** (Q2 is 2) **or** control of confounders was not described (Q1 is 3) **and** (Q2 is 4).  **Weak:** will be assigned when less than 60% of relevant confounders were controlled (Q1 is 1) **and** (Q2 is 3) |
| **E) DATA COLLECTION METHODS** | |
| **(Q1) Were data collection tools for BF shown to be valid?**   1. 1. Yes 2. 2. No 3. 3. Can’t tell | Tools for primary outcome measures must be described as reliable and valid. If ‘face’ validity or ‘content’ validity has  been demonstrated, this is acceptable. Some sources from which data may be collected are described below:  Self reported data includes data that is collected from participants in the study (e.g. completing a questionnaire,  survey, answering questions during an interview, etc.).  Assessment/Screening includes objective data that is retrieved by the researchers. (e.g. observations by  investigators).  Medical Records/Vital Statistics refers to the types of formal records used for the extraction of the data.  **Reliability and validity can be reported in the study or in a separate study. For example, some**  **standard assessment tools have known reliability and validity.** |
| **(Q2) Were data collection tools for BF shown to be reliable?**   1. 1. Yes 2. 2. No 3. 3. Can’t tell |  |
| \| **RATE THIS SECTION** \| **STRONG** \| **MODERATE** \| **WEAK** \| \| --- \| --- \| --- \| --- \| \| See dictionary \| 1 \| 2 \| 3 \| | **Strong: :** The data collection tools have been shown to be valid (Q1 is 1); **and** the data collection tools have been shown to be reliable (Q2 is 1).  **Moderate:** The data collection tools have been shown to be valid (Q1 is 1); **and** the data collection tools have not been  shown to be reliable (Q2 is 2) **or** reliability is not described (Q2 is 3).  **Weak:** The data collection tools have not been shown to be valid (Q1 is 2) **or** both reliability and validity are not  described (Q1 is 3 and Q2 is 3). |
| **F) WITHDRAWALS AND DROP-OUTS** | |
| **(Q1) Were withdrawals and drop-outs reported in terms of numbers and/or reasons per group?**   1. 1. Yes 2. 2. No 3. 3. Can’t tell 4. 4. Not Applicable (i.e. one time surveys) | Score **YES** if the authors describe BOTH the numbers and reasons for withdrawals and drop-outs.  Score **NO** if either the numbers or reasons for withdrawals and drop-outs are not reported.  Score **NOT APPLICABLE** if the study was a one-time interview or survey where there was not follow-up data reported.  The percentage of participants completing the study refers to the % of subjects remaining in the study at the final data  collection period in all groups (i.e. control and intervention groups). |
| **(Q2) Indicate the percentage of participants completing the study. (If the percentage differs by groups, record the lowest).**   1. 1. 80 -100% 2. 2. 60 - 79% 3. 3. less than 60% 4. 4 Can’t tell 5. 5. Not Applicable (i.e. Retrospective case-control) |  |
| \| **RATE THIS SECTION** \| **STRONG** \| **MODERATE** \| **WEAK** \|  \| \| --- \| --- \| --- \| --- \| --- \| \| **See dictionary** \| 1 \| 2 \| 3 \| Not Applicable \| | **Strong:** will be assigned when the follow-up rate is 80% or greater (Q1 is 1 and Q2 is 1).  **Moderate:** will be assigned when the follow-up rate is 60 – 79% (Q2 is 2) **OR** Q1 is 4 or Q2 is 5.  **Weak:** will be assigned when a follow-up rate is less than 60% (Q2 is 3) or if the withdrawals and drop-outs were not  described (Q1 is No or Q2 is 4).  **Not Applicable:** if Q1 is 4 or Q2 is 5. |
| **H) ANALYSES (**Was the quantitative analysis appropriate to the research question being asked?**)** | |
| **(Q1) Indicate the format of the results on the BF?**   1. Crude prevalence 2. Prevalence adjusted for confounders 3. Crude mean 4. Mean adjusted for confounders | |
| **GLOBAL RATING** | |
| **STRONG**  **MODERATE**  **WEAK** | **STRONG:** no WEAK ratings  **MODERATE:** one WEAK rating  **WEAK:** two and more WEAK ratings |

**Note.** This tool with its dictionary was adapted from the quality assessment tool for quantitative studies of the Effective Public Health Practice Project^18^.

**Supplementary Table 3. Transformation mean and standard deviation**

| **Target Likert-type** | | **Original Likert-type** | | | | |
| --- | --- | --- | --- | --- | --- | --- |
|  |  | **0 to 3** | **0 to 6** | **0 to 4** | **1 to 3** | **1 to 4** |
| **1 to 5** | **M_t_** | (M+1)*5/4 | (M+1)*5/7 | M+1 | M*5/3 | M*5/4 |
|  | **SD_t_** | SD*5/4 | SD*5/7 | SD | SD*5/3 | SD*5/4 |

**Supplementary Table 4. Transformation of mean and standard deviation in subgroups**

| Target | subgroup 1 | subgroup 2 | subgroup i | 2 Groups | i Groups |
| --- | --- | --- | --- | --- | --- |
| N_t_ | n_1_ | n_2_ | n_i_ | n_1_+n_2_ | n_1_+n_2_...+n_i_ |
| M_t_ | M_1_ | M_2_ | M_i_ | (n_1_M_1_+n_2_M_2_)/(n_1_+n_2_) | $\frac{\sum Mini}{\sum ni}$ |
| SD_t_ | SD_1_ | SD_2_ | SD_i_ | $\sqrt{\frac{\left( n1-1 \right){SD1}^{2}+\left( n2-1 \right){SD2}^{2}+\frac{n1n2}{n1+n2}({M1}^{2}+{M2}^{2}-2M1M2)}{n1+n2-1}}$ | $\sqrt{\frac{\sum\left[ \left( {SDi}^{2}+Mi2 \right)ni \right]}{\sum ni}- \left( \frac{\sum Mini}{\sum ni} \right)}$^2^ |

**Supplementary Table 5. Detailed studies reported mean BF (n=55)- Please refer to the xlsxl file**

**Supplementary Table 6. Subgroup analysis-without stratification**

| **Subgroup** | **No. Studies/ Estimates pooled** | | **No. Survivors** | **Mean (95% CI)*** | **I^2^ (%)** | **Differences between subgroups (p)*** |
| --- | --- | --- | --- | --- | --- | --- |
|  | **No. Studies** | **No. Estimates pooled** |  |  |  |  |
| **BF mean level** | **55** | **56** | **25972** | **3.25(3.11, 3.40)** | **99.5** |  |
| Study design |  |  |  |  |  | χ^2^ = 6.56, p **= 0.01** |
| Longitudinal | 20 | 20 | 7967 | 3.48 (3.29, 3.66) | 99.5 |  |
| Cross-sectional | 35 | 36 | 18005 | 3.13 (2.94, 3.32) | 99.5 |  |
| Methodological quality |  |  |  |  |  | χ^2^ = 2.46, p = 0.29 |
| Strong | 2 | 2 | 415 | 3.17 (2.76, 3.58) | 87.4 |  |
| Moderate | 18 | 18 | 5588 | 3.09 (2.81, 3.38) | 99.6 |  |
| Weak | 35 | 36 | 19969 | 3.34 (3.18, 3.51) | 99.4 |  |
| Time since diagnosis ^0^ |  |  |  |  |  | χ^2^ = 2.36, p = 0.50 |
| < 1 year | 9 | 9 | 4235 | 3.18 (2.97, 3.57) | 99.7 |  |
| 1- 5 years | 16 | 17 | 6266 | 3.37 (3.18, 3.56) | 99.2 |  |
| > 5 years | 11 | 11 | 9971 | 3.36 (3.11, 3.60) | 97.6 |  |
| Not given | 19 | 19 | 5500 | 3.13 (2.82, 3.43) | 99.6 |  |
| Gender |  |  |  |  |  | χ^2^ = 3.51, p = 0.17 |
| Female | 14 | 15 | 4324 | 3.13 (2.77, 3.49) | 99.7 |  |
| Male | 4 | 5 | 3114 | 2.96 (2.51, 3.40) | 98.0 |  |
| Both | 37 | 36 | 18534 | 3.35 (3.20, 3.50) | 99.2 |  |
| Mean age at survey ^0^ |  |  |  |  |  | χ^2^ = 23.11, **p < 0.01** |
| ≤ 50 | 18 | 19 | 5362 | 3.03 (2.76, 3.29) | 99.7 |  |
| 51-60 | 18 | 18 | 4697 | 3.58 (3.43, 3.74) | 97.3 |  |
| 61-70 | 12 | 12 | 14363 | 3.44 (3.29, 3.58) | 98.9 |  |
| >70 | 4 | 4 | 795 | 2.57 (2.01, 3.14) | 99.0 |  |
| Not given | 3 | 3 | 755 | 2.91 (2.12, 3.69) | 99.7 |  |
| **BF prevalence**^#^ | **12** | | **10720** | **0.97 (0.94, 1.00)** | **94.1** |  |
| Study design |  | |  |  |  | χ^2^ = 0.89, p = 0.35 |
| Longitudinal | 3 | | 1444 | 0.92 (0.78, 1.00) | 97.8 |  |
| Cross-sectional | 9 | | 9276 | 1.00 (0.99, 1.00) | 91.2 |  |
| Methodological quality |  | |  |  |  | χ^2^ = 0.88, p = 0.35 |
| Strong | 0 | |  |  |  |  |
| Moderate | 6 | | 1890 | 0.96 (0.89, 1.00) | 94.7 |  |
| Weak | 6 | | 8830 | 1.00 (0.99, 1.00) | 94.6 |  |
| Time since diagnosis ^0^ |  | |  |  |  | χ^2^ = 1.29, p = 0.53 |
| ≤ 5years | 3 | | 1781 | 1.00 (1.00, 1.00) | 0 |  |
| > 5 years | 5 | | 8308 | 0.95 (0.86, 1.00) | 97.0 |  |
| Not given | 4 | | 631 | 1.00 (0.99, 1.00) | 39.7 |  |
| Mean age at survey ^0^ |  | |  |  |  | χ^2^ = 44.70, **p < 0.01** |
| ≤ 50 | 6 | | 2078 | 0.96 (0.89, 1.00) | 94.4 |  |
| 51-60 | 3 | | 1265 | 1.00 (1.00, 1.00) | 0 |  |
| >60 | 3 | | 7377 | 0.99 (0.98, 0.99) | 48.7 |  |
| Geographic origin |  | |  |  |  | χ^2^ = 47.58, **p < 0.01** |
| North America^2^ | 4 | | 776 | 1.00 (1.00, 1.00) | 0 |  |
| Asia^3^ | 3 | | 1781 | 1.00 (1.00, 1.00) | 0 |  |
| Australia | 2 | | 786 | 0.88 (0.69, 1.00) | 98.9 |  |
| Europe^4^ | 3 | | 7377 | 0.99 (0.98, 0.99) | 48.7 |  |
| **MODERATE-TO-HIGH BF prevalence**^#^ | **14** | | **12363** | **0.74 (0.61, 0.85)** | **96.3** |  |
| Study design |  | |  |  |  | χ^2^ = 7.51, **p < 0.01** |
| Longitudinal | 2 | | 488 | 0.56 (0.52, 0.60) | 0 |  |
| Cross-sectional | 12 | | 11875 | 0.77 (0.63, 0.86) | 98.0 |  |
| Methodological quality |  | |  |  | - | χ^2^ = 2.34, p = 0.13 |
| Moderate | 3 | | 1079 | 0.64 (0.56, 0.71) | 89.1 |  |
| Weak | 11 | | 11284 | 0.77 (0.61, 0.87) | 98.1 |  |
| Time since diagnosis ^0,1^ |  | |  |  |  | χ^2^ = 0.65, p = 0.72 |
| ≤ 5years | 3 | | 1193 | 0.81 (0.62, 0.92) | 96.0 |  |
| > 5 years | 6 | | 8365 | 0.70 (0.45, 0.87) | 96.6 |  |
| Not given | 5 | | 2805 | 0.74 (0.58, 0.86) | 96.9 |  |
| Mean age at survey ^0^ |  | |  |  |  | χ^2^ = 15.86, **p < 0.01** |
| ≤ 50 | 3 | | 973 | 0.83 (0.48, 0.96) | 98.1 |  |
| 51-60 | 6 | | 1600 | 0.82 (0.72, 0.89) | 92.6 |  |
| >60 | 4 | | 9690 | 0.53 (0.40, 0.66) | 98.7 |  |
| Not given | 1 | | 100 | 0.59 (0.49, 0.69) |  |  |
| Geographic origin |  | |  |  |  | χ^2^ = 31.53, **p < 0.01** |
| North America^2^ | 3 | | 480 | 0.78 (0.68, 0.86) | 82.6 |  |
| Asia^3^ | 4 | | 1635 | 0.91 (0.81, 0.96) | 96.9 |  |
| Australia | 2 | | 458 | 0.56 (0.52, 0.61) | 29.7 |  |
| Europe^4^ | 5 | | 9790 | 0.54 (0.43, 0.65) | 98.2 |  |

* Random effect model

^0^ These categories were arbitrarily determined.

^1^ Time since diagnosis < 1 year: n=1, 1-5 year: n=2, > 5 years: n=6, not given: n=3.

^2^ USA and Canada

^3^ China and Japan

^4^ Germany, Spain, and UK

**Supplementary figure 1. Quality assessment of included studies (n=55)**

**
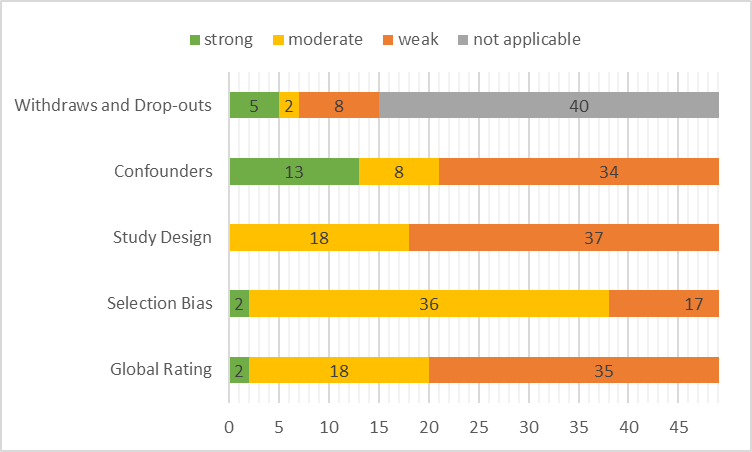
**

**Supplementary figure 2. Sensitivity analysis**
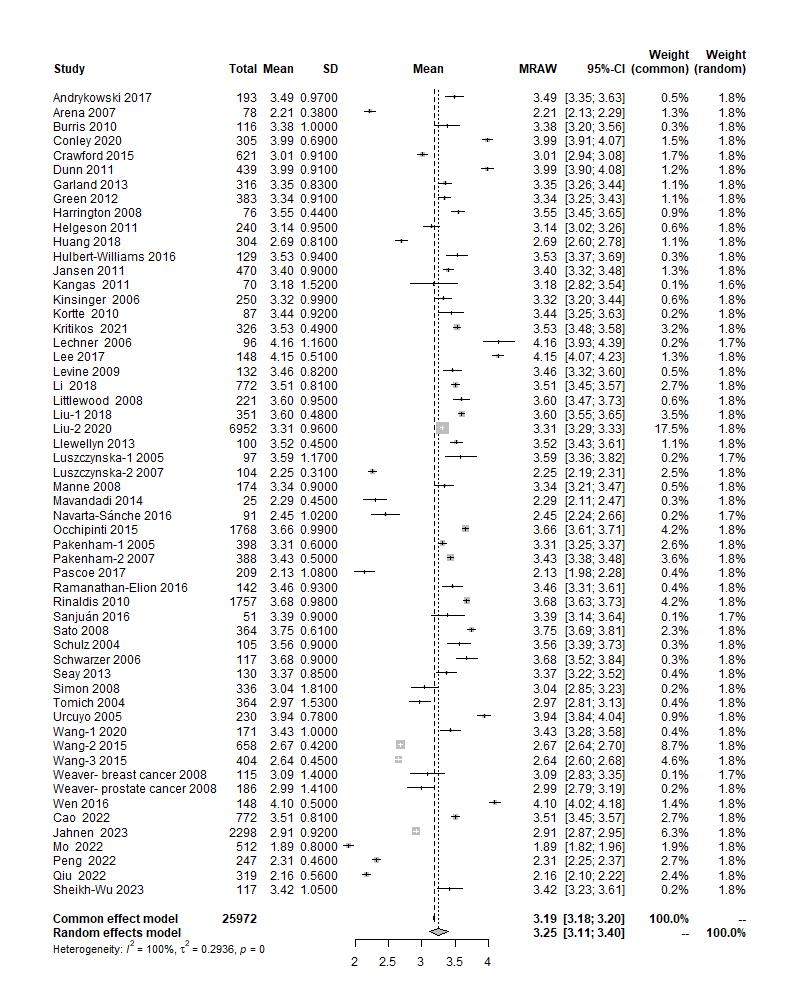

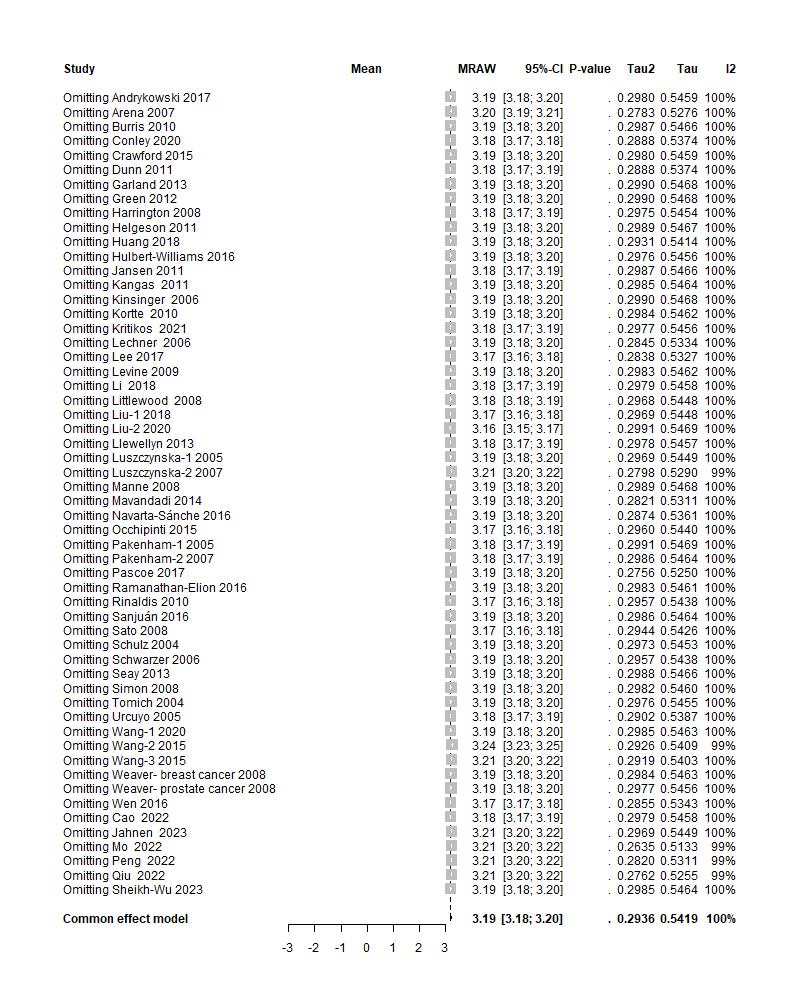


A-BF mean (n=56)


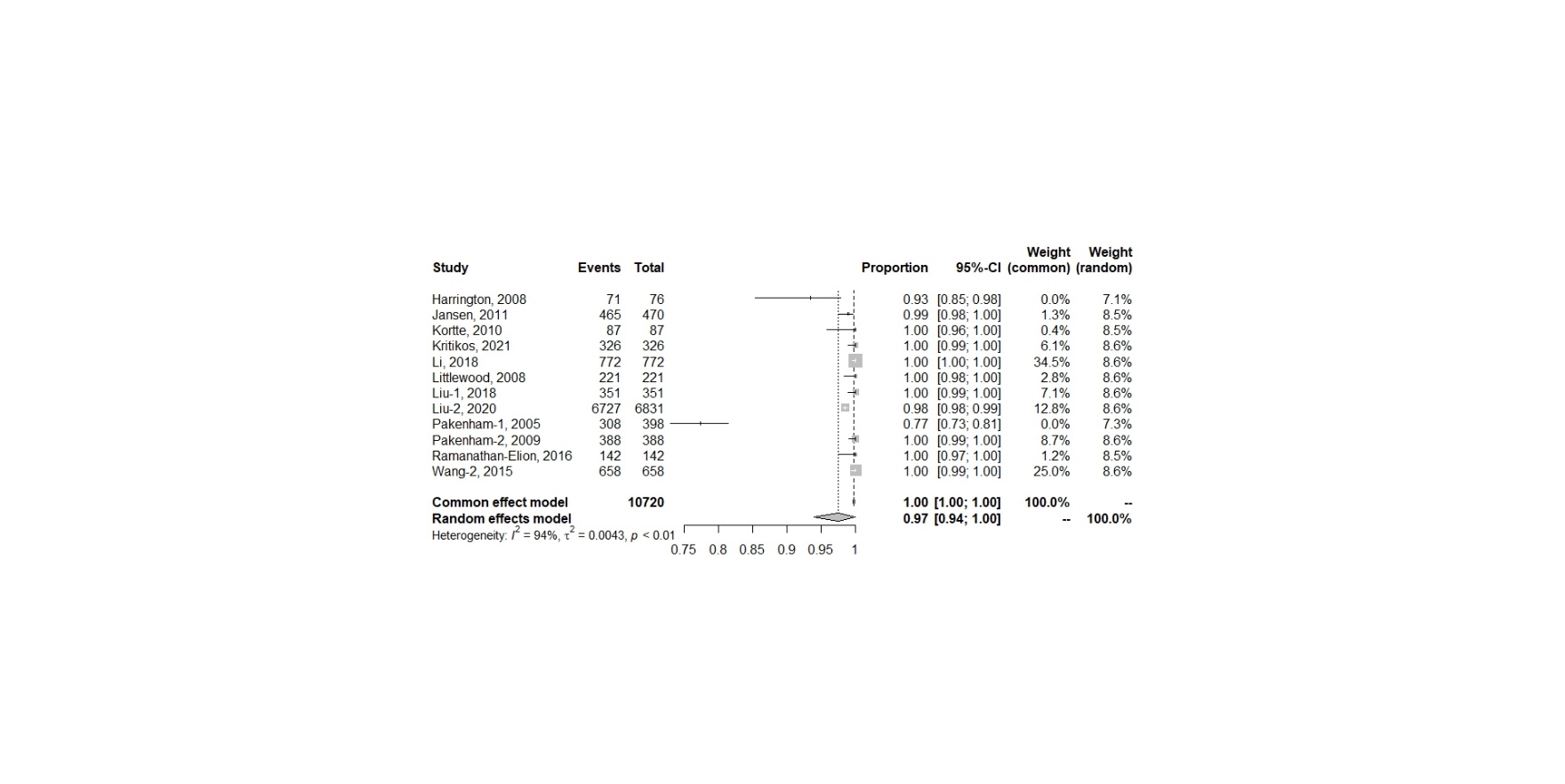

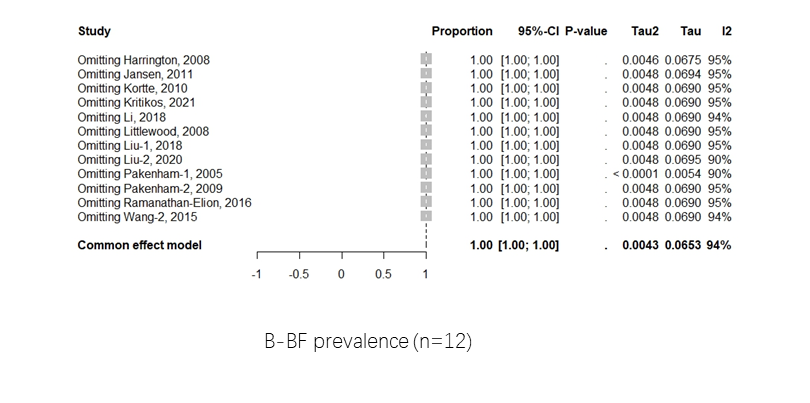


B- BF prevalence (n=12)


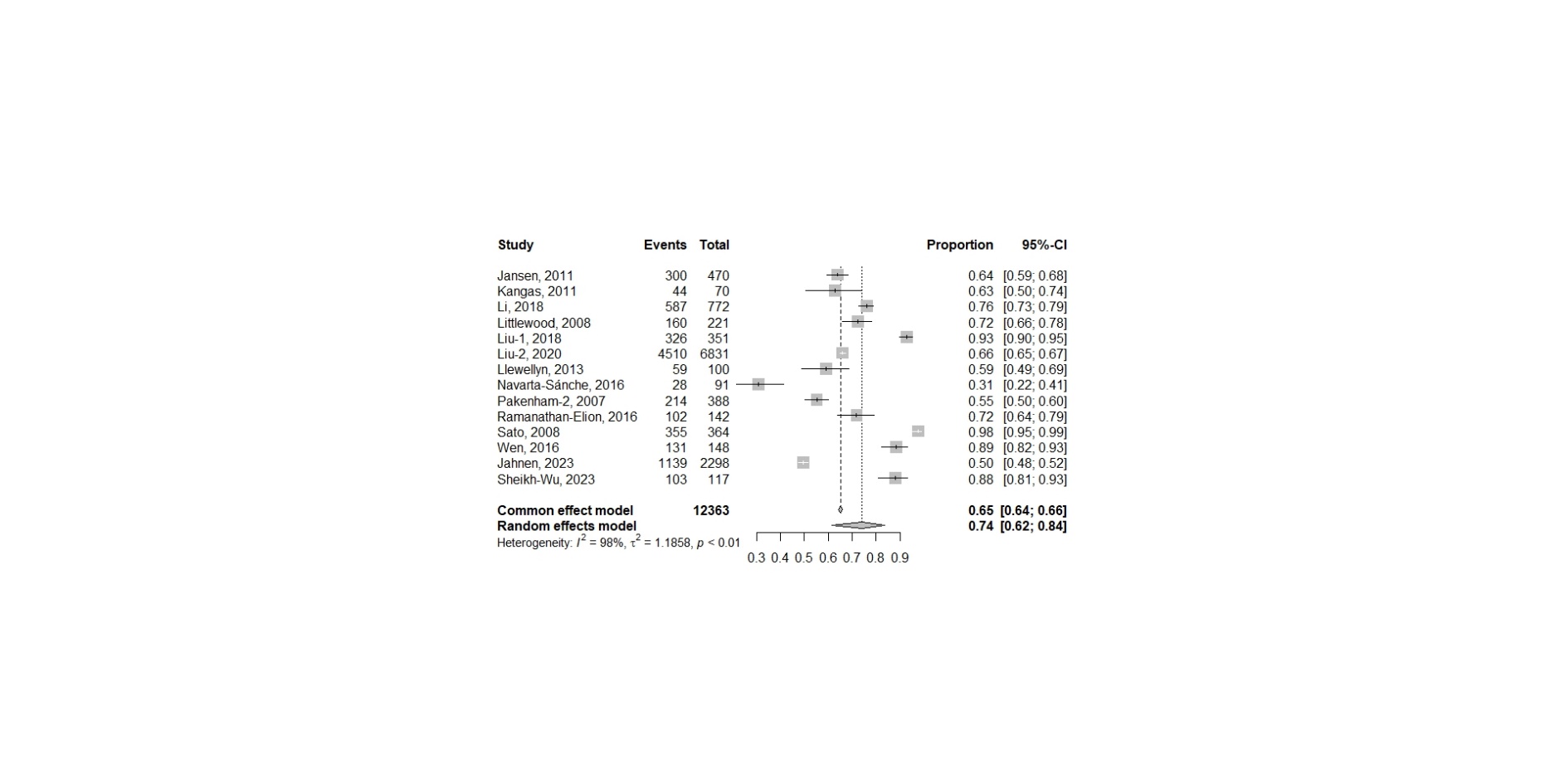

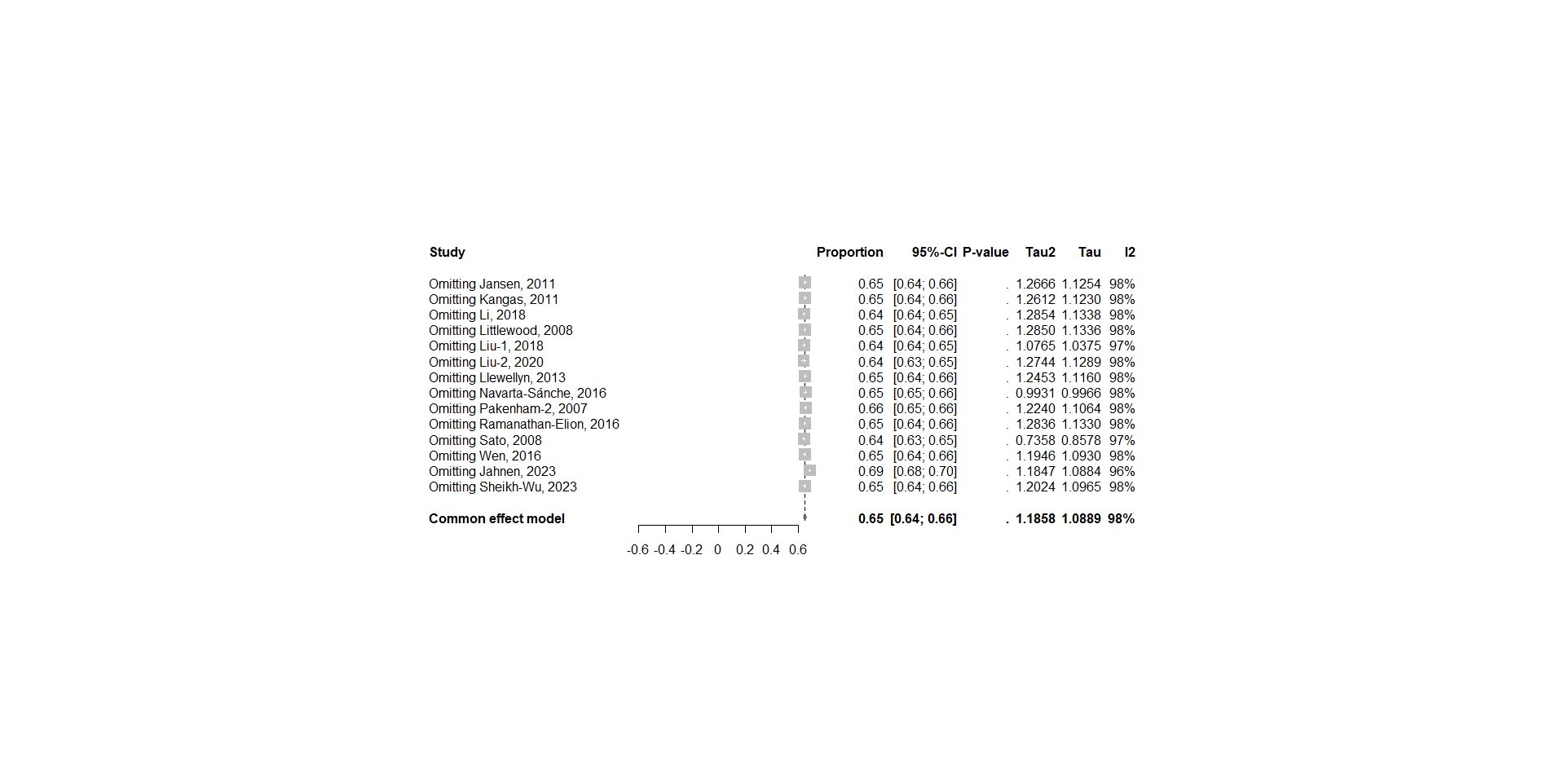


C- MODERATE-TO-HIGH BF prevalence (n=14)

**Supplementary figure 3. funnel plots**


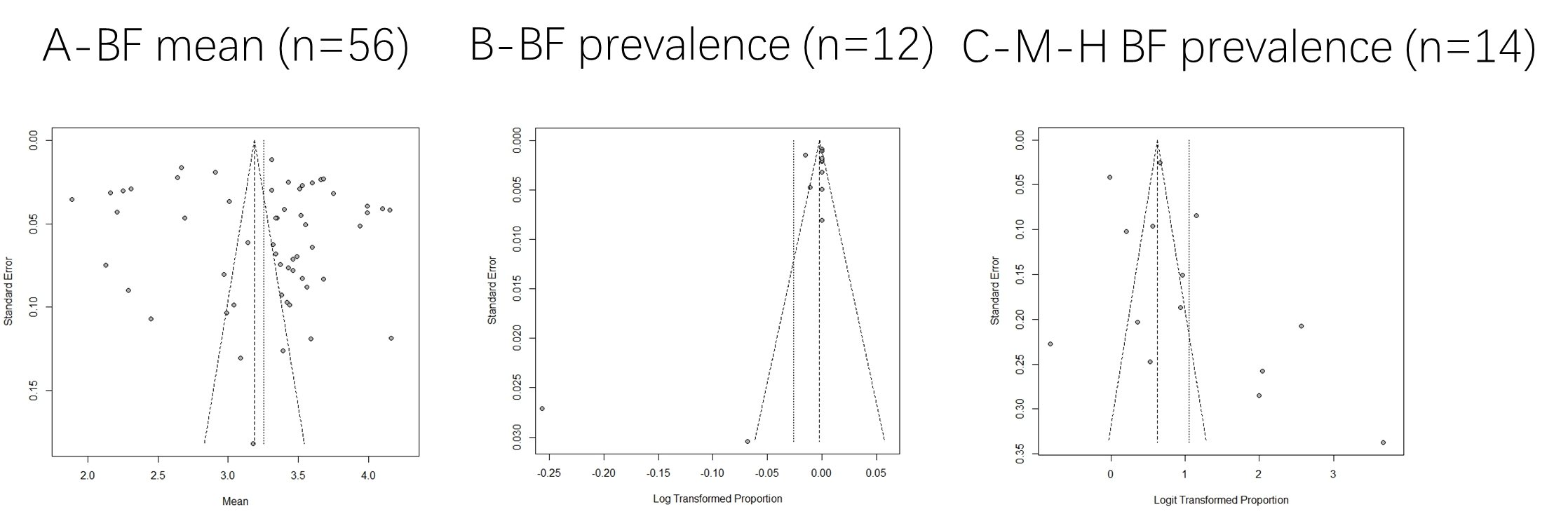

Supplement: Supplementary file 1 [file DataSheet1.docx]
